# Oral magnesium supplementation improves glycemic control and lipid profile in children with type 1 diabetes and hypomagnesaemia

**DOI:** 10.1097/MD.0000000000006352

**Published:** 2017-03-24

**Authors:** Doaaa Shahbah, Tamer Hassan, Saeed Morsy, Hosam El Saadany, Manar Fathy, Ashgan Al-Ghobashy, Nahla Elsamad, Ahmed Emam, Ahmed Elhewala, Boshra Ibrahim, Sherief El Gebaly, Hany El Sayed, Hanan Ahmed

**Affiliations:** a Department of Pediatrics; b Department of Clinical Pathology, Zagazig University, Zagazig, Egypt.

**Keywords:** children, diabetes, lipid, magnesium, supplementation

## Abstract

Dietary supplementation with magnesium (Mg) in addition to classical therapies for diabetes may help in prevention or delaying of diabetic complications.

We aimed to evaluate the status of serum Mg in children with type 1 diabetes and assessing its relationship to glycemic control and lipid profile. Then evaluating the effect of oral Mg supplementation on glycemic control and lipid parameters.

We included 71 children at Pediatric Endocrinology Outpatient Clinic, Zagazig University, Egypt with type 1 diabetes and assessed HBA1c, lipid profile, and serum Mg at the start of study. Patients with serum Mg level < 1.7 mg/dL were given 300 mg Mg oxide for 3 months. After that we reevaluated HBA1c, lipid profile, and serum Mg in all patients.

The study included 71 patients with type 1 diabetes (32 males and 39 females); their mean age was 9.68 ± 3.99 years. The mean serum Mg level was 1.83 ± .27 mg/dL. Hypomagnesemia was detected in 28.2% study patients. Serum Mg was found to be positively correlated with high density lipoprotein, mean corpuscular volume and platelet count (*P* < 0.001), and negatively correlated with age, HbA1c, triglycerides, total cholesterol, low density lipoprotein, and duration of diabetes (*P* < 0.001). There was significant reduction in HBA1c in group given Mg supplementation. HBA1c was initially 10.11% ± 0.87%. After 3 months of oral Mg supplementation it is reduced to 7.88% ± 0.42% (*P* < 0.001). There was statistically significant difference in lipid parameters in hypomagnesemic diabetic patients before and after Mg supplementation with significant reduction in serum triglycerides, LDL, and total cholesterol following Mg supplementation with *P* < 0.001. Although HDL shows a significant increase after Mg supplementation in hypomagnesemic diabetic children with *P* < 0.001.

Correction of hypomagnesemia in type 1 diabetic children with oral Mg supplements is associated with optimization of glycemic control and reduction of atherogenic lipid fraction as well as increase in protective lipid fraction.

## 1 Introduction

Increasing attention has been given to the role of certain elements in the pathogenesis of diabetes mellitus and in the progression of its complications.^[1]^

Diabetes mellitus has been suggested to be the most common metabolic disorder associated with magnesium (Mg) deficiency, having 25% to 39% prevalence.^[2]^ Numerous causes for low Mg levels in diabetics can be listed including diets low in Mg, osmotic diuresis that leads to high renal excretion of Mg, insensitivity to insulin that affects intracellular Mg transport and causes increased loss of extracellular Mg, usage of loop and thiazide diuretics that promote Mg wasting, diabetic autonomic neuropathies, and reduced tubular reabsorption due to insulin resistance. Additionally, continuous Mg deficiency correlates to higher levels of TNFα, which may also contribute to postreceptor insulin resistance.^[3]^

Such Mg deficits have been linked to the development of atherosclerosis,^[4,5]^ and in patients with coronary atherosclerosis, a Mg deficit has been related to an atherogenic lipid profile.^[6]^ Therefore, Mg deficit in patients with type 1diabetes could be associated with the development of late diabetic complications, especially macroangiopathy.^[7]^

There are challenges in diagnosing Mg deficiency because of its distribution in the body. Mg is an intracellular cation and its blood concentrations may not accurately mirror Mg status.^[8]^ However, reductions in normal serum Mg concentrations (1.8–2.3 mg/dL) signify deficiency. Therefore, serum Mg concentrations are specific, but not sensitive, to Mg deficiency.^[9]^ There are other methods to assess Mg status, but the serum level is the most common and practical test in the clinical setting.^[10]^

Dietary supplementation with Mg in addition to classical therapies for diabetes may help in prevention or delaying of diabetic complication. Oral Mg supplementation improves insulin sensitivity and metabolic control in type 2 diabetics with lower serum Mg levels. It has also been shown that it has beneficial effect on lipid profile of diabetic patients.^[11]^

Several studies were focused on evaluating Mg status in patients with type 2 diabetes and on role of Mg supplementation in prevention of diabetic complications and optimization of diabetic control. However, few studies have been concerned with this issue in children with type 1 diabetes with opposing results.

We aimed to evaluate the status of serum Mg in children with type 1 diabetes and assessing its relationship to glycemic control and lipid profile. Then evaluating the effect of oral Mg supplementation on glycemic control and lipid parameters.

## 2 Methods

A prospective cohort study in type 1 diabetic children was conducted at Pediatric Endocrinology Outpatient Clinic, Zagazig University, Egypt during the period from February 2014 to December 2014.

### 2.1 Inclusion criteria

Subjects who met the following criteria were consecutively enrolled during their follow-up at Pediatric Endocrinology Outpatient Clinic, Zagazig University.

(1) Type 1 diabetes
(2) Age between 1 and 18 years.
(3) Both sexes

### 2.2 Exclusion criteria

(1) Children who have renal disease detected by serum urea and creatinine test
(2) Diuretics usage in the last 2 weeks.
(3) Children with persistent diarrhea and vomiting.

All subjects underwent the following:

(1) Thorough history taking and complete physical examination.
(2) Routine investigations according to our local standards every 3 months, for example, complete blood count, renal function tests, random blood glucose, glycosylated hemoglobin, and so on.
(3) Special investigations:
  (a) Serum Mg level by Integra 400 Plus (Roche, Germany)
  (b) Lipid profile by Integra 400 Plus (Roche, Germany).

First samples were collected from all patients for the evaluation of serum Mg, HbA1c, and lipid profile. Based on serum Mg level we divided patients into 2 groups.

- Normomagnesemic group (group II) with serum Mg ≥ 1.7 mg/dL and
- Hypomagnesemic group (group I) with serum Mg < 1.7 mg/dL.^[12]^

The hypomagnesemic group (serum Mg < 1.7 mg/dL) were given oral Mg supplementation at a dose of 300 mg of Mg oxide for 3 months^[10]^ (Mg plus tablets, Mepaco-Medifood, Sharkeya, Egypt).

At the end of 3 months, other samples were collected from all patients for the reevaluation of serum Mg, HbA1c, and lipid profile.

### 2.3 Ethics

The protocol developed is according to Declaration of Helsinki 1964, as revised in 2000, and was approved by the institutional review board at our faculty. Informed consent was obtained from all patients’ guardians prior to participation.

### 2.4 Statistical analysis

All data were collected, tabulated, and statistically analyzed using SPSS 15.0 for windows (SPSS Inc., Chicago, IL). Continuous Quantitative variables, for example, age were expressed as the mean ± SD and median (range), and categorical qualitative variables were expressed as absolute frequencies “number” and relative frequencies (percentage).

All values are expressed as median and range or mean ± SD. Differences between groups were assessed by paired Student *t* test or the Mann–Whitney *U* test. Correlation between variables was assessed using Spearman rank correlation coefficient. A *P* value of <0.05 was considered significant.

## 3 Results

The study included 71 patients with type 1 diabetes (32 males and 39 females); their mean age was 9.68 ± 3.99 years. The mean serum Mg level was 1.83 ± 0.27 mg/dL. Taking cut-off level of serum Mg <1.7 mg/dL for the definition of hypomagnesemia,^[12]^ hypomagnesemia was detected in 28.2% study patients.

### 3.1 Correlations between serum magnesium and some study parameters

Serum Mg was found to be positively correlated with high density lipoprotein (HDL), mean corpuscular volume (MCV) and platelet count (*P* < 0.001), and negatively correlated with age, HbA1c, triglycerides, total cholesterol, low density lipoprotein (LDL), and duration of diabetes (*P* < 0.001) (Table 1). Characteristics of patients with or without hypomagnesemia are displayed in Table 2.

**Table 1:**
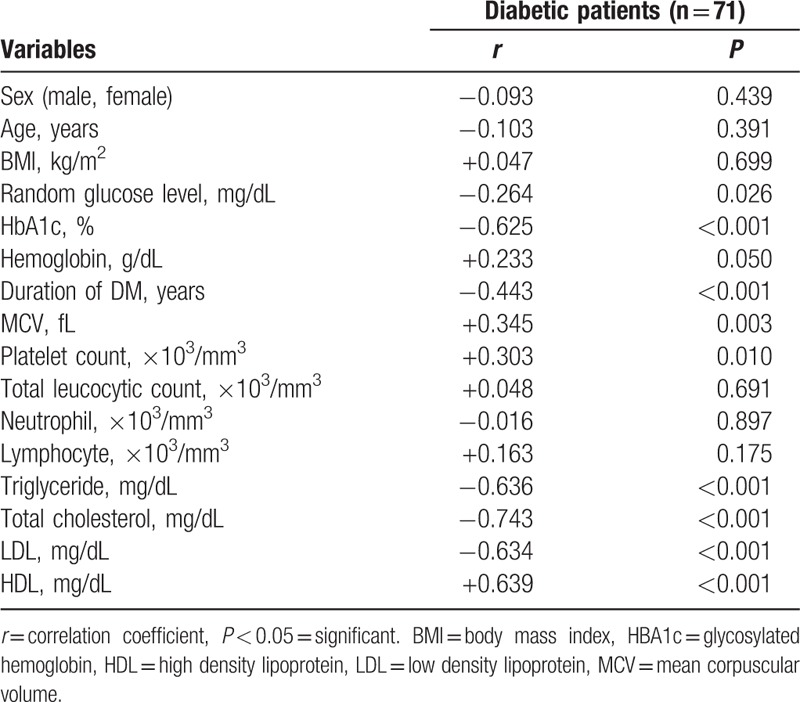
Correlations between serum magnesium level and some study parameters in diabetic patients.

**Table 2:**
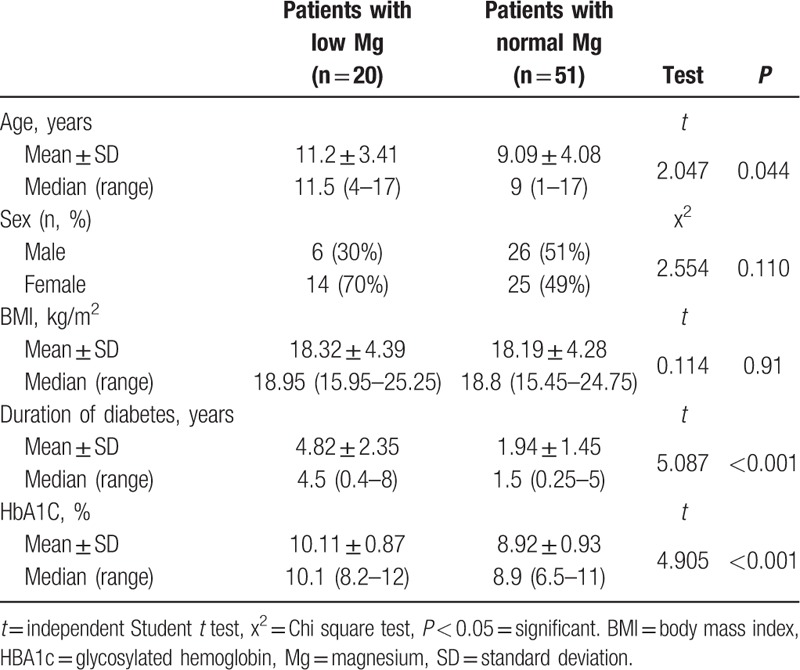
Characteristics of patients with and without hypomagnesemia.

### 3.2 Relation of serum magnesium to duration of diabetes

There was statistically significant difference in duration of diabetes between the hypomagnesemic and normomagnesemic groups, as mean ± SD of diabetes duration was 4.8 ± 2.39 years in hypomagnesemic group versus 1.94 ± 1.45 years in normomagnesemic group with *P* < 0.001.

### 3.3 Relation of serum magnesium to age of patients

The age was statistically different between normomagnesemic and hypomagnesemic patients, being higher in hypomagnesemic one with mean ± SD was 10.11 ± 0.87 years versus 9.03 ± 3.51 years in normomagnesemic group, while mean age of diagnosis was 4.21 ± 2.19 with no significant difference between normomagnesemic and hypomagnesemic groups.

### 3.4 Relation of serum magnesium to glycemic control

There was statistically significant difference in HbA1c between hypomagnesemic group and normomagnesemic groups with higher value of HbA1c in low Mg group, as mean ± SD was 11.93% ± 3.17% in group I versus 8.92% ± 0.93% in group II.

### 3.5 Relation of serum magnesium to lipid profile

There was statistically significant difference between normal and hypomagnesemic diabetic children with lower value of HDL in hypomagnesemic group and higher values of the rest of lipid parameters before Mg supplementation (*P* < 0.001) (Table 3).

**Table 3:**
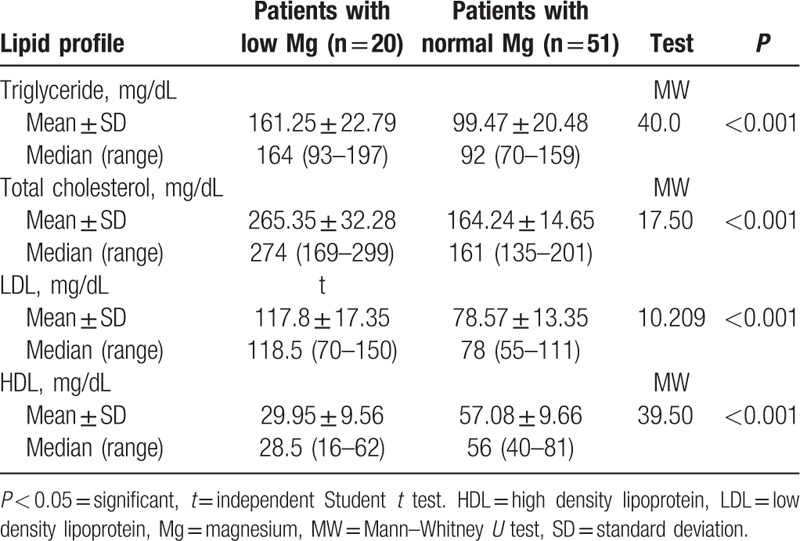
Lipid profile in diabetic patients at the start of study.

### 3.6 Potential predictors of serum magnesium level in diabetic children

There are many factors affecting serum Mg level in diabetic children, including age, sex, and duration of diabetes (Table 4). The most contributing factor to hypomagnesemia was the duration of diabetes which exhibited statistically high significant difference (*P* < 0.001).

**Table 4:**
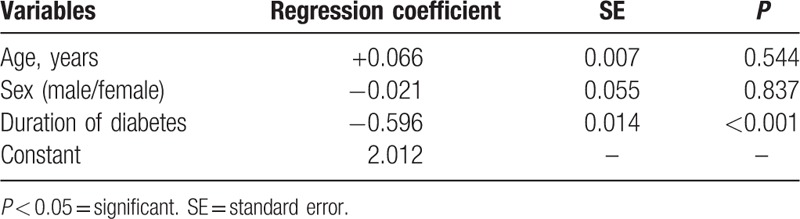
Multivariate linear regression of potential predictors of serum magnesium level in diabetic children.

### 3.7 Effect of oral magnesium supplementation on serum magnesium, glycemic control, and lipid profile

There was statistically significant difference in serum Mg level in diabetic children with hypomagnesemia before and after Mg supplementation with significant increase in serum Mg following supplementation with Mg (mean value of serum Mg before [1.45 ± 0.15] became [1.94 ± 0.12]) after (*P* < 0.001).

There was statistically significant difference in HbA1c between hypomagnesemic and normomagnesemic groups with higher value of HbA1c in low Mg group (group I) before Mg supplementation, as mean ± SD was 10.11 ± 0.87 in group I versus 8.92 ± 0.93 in group II. After Mg supplementation to group I, there was statistically significant difference with lower value of HbA1c in group I (mean ± SD, 7.88 ± 0.42) than it in group II (mean ± SD, 8.99 ± 0.83). There was significant reduction in HBA1c in group given Mg supplementation. HBA1c was 10.11% ± 0.87% before supplementation. After 3 months of oral Mg supplementation it reduced to 7.88% ± 0.42% (*P* < 0.001) (Table 5).

**Table 5:**
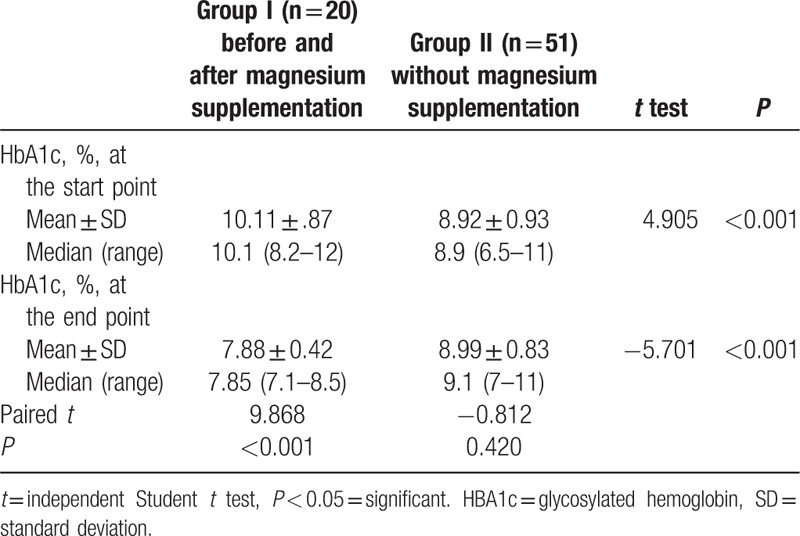
HBA1c in diabetic patients at the start and the end of study.

There was statistically significant difference in lipid parameters in hypomagnesemic diabetic patients before and after Mg supplementation with significant reduction in serum triglycerides, LDL, and total cholesterol following Mg supplementation with *P* < 0.001. Although HDL shows a significant increase after Mg supplementation in hypomagnesemic diabetic children with *P* < 0.001 (Table 6).

**Table 6:**
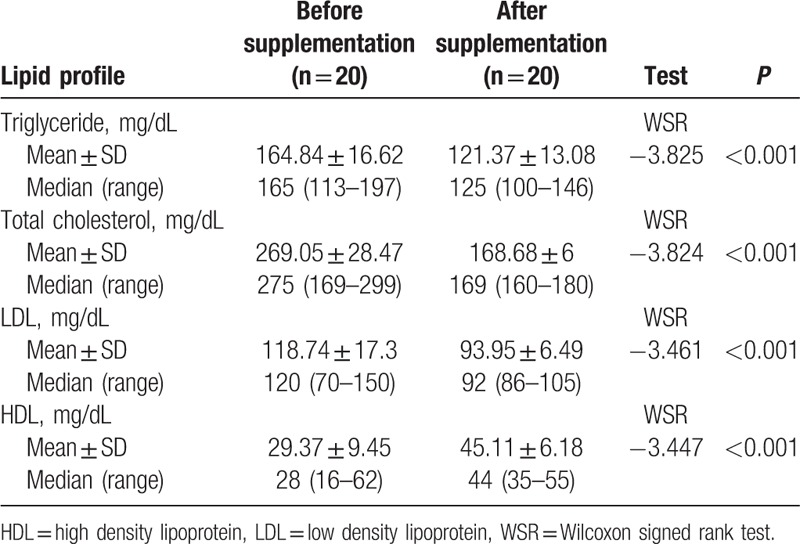
Lipid profile changes in hypomagnesemic patients with magnesium supplementation.

## 4 Discussion

Type 1 diabetes mellitus (T1DM) is the most life threatening endocrine disorder of children and its incidence appears to be increasing.^[13]^

Diabetes mellitus is the most chronic disease studied with respect to serum Mg level. Mg plays a significant role in glucose and insulin metabolism, mainly through its impact on tyrosine kinase enzyme, Mg may directly affect glucose transporter protein activity 4 and regulate glucose translocation into the cell.^[14]^

Serum Mg level has been assessed in type 2 diabetes mellitus (T2DM) in different studies. However, there are few studies, concerned with serum Mg and its relation to diabetic control in type 1 diabetes. Among these studies, there was a great controversy in results regarding frequency of hypomagnesemia and correlation between Mg level and HbA1c. Some studies have tried Mg supplementation for hypomagnesemia in type 2 diabetes as a trial to improve diabetic control and reduce diabetic complications. However, to the best of our knowledge, this is the 1st study in which Mg supplementation has been tried in hypomagnesemic type 1 diabetic children to assess its role in glycemic and lipid control.

In our study, we observed a negative correlation between serum Mg level and HbA1c. This agreed with Galli-Tsinopoulou et al^[12]^ on Athens type 1 diabetic children that showed a lower Mg level in patients with poor glycemic control with high HbA1c. Ramadass et al,^[15]^ Sinha and Sen^[16]^ showed similar results in adult patients with type 2 diabetes. Also Mikhail and Ehsanipoor^[17]^ observed an inverse correlation between total Mg and HbA1c.

Inconsistent with our result, Lin et al^[18]^ and Salmonowicz et al^[19]^ did not show any correlation between serum Mg level and HbA1c in type 1 diabetic children and adolescents. Also Matthiesen et al^[20]^ did not observe any relationship between HbA1c and ionized Mg, and Wegner et al^[21]^ failed to show any relation between serum Mg and glycemic controls in the form of fasting blood glucose.

There was statistically significant difference in serum lipids level between our study groups with higher triglycerides, total cholesterol, and LDL with lower HDL in group with hypomagnesemia compared to those with normal serum Mg. A negative correlation is noted between serum Mg and triglycerides, total cholesterol, and LDL. However, there was a positive correlation between serum Mg and HDL.

These results are in concordance with Mishra et al^[22]^ study on adult type 2 diabetic patients that revealed a negative correlation between serum Mg and triglycerides and a positive correlation with HDL, but did not show any significant correlation between total cholesterol or LDL with serum Mg. Moreover, Srinivasan et al^[23]^ revealed a negative correlation between serum Mg and triglycerides especially in poorly controlled diabetics, and Rasheed et al^[24]^ revealed a positive correlation of serum Mg with HDL, but there was a nonsignificant correlation with other lipid parameters.

Guerrero-Romero and Rodríguez-Morán^[8]^ showed that in patients with T2DM, hypomagnesemia is linked with low levels of HDL, irrespective of serum glucose level.

Our results are inconsistent with Wegner et al^[21]^ on type 1 diabetic children that found that there was no significant difference in lipid parameters between patients with normal and those with low serum Mg level. Also, Xu et al^[25]^ failed to show any association between serum Mg and lipid profile in diabetic patients with or without complications.

In our study, we detected a negative correlation between serum Mg level in diabetic children with duration of diabetes. This agrees with the study done by Shaikh et al^[26]^ on type 1 and 2 diabetic patients who detected hypomagnesemia in 36% of patients with 3 to 5 years duration of diabetes versus 71% of patients with 6 to 10 years duration of diabetes and 72% of patients with 11 to 15 years duration of diabetes. This is also consistent with a study on adults with T2DM by Mishra et al^[22]^ that showed a negative correlation between serum Mg level and duration of diabetes (*r* = −0.789). However, Lin et al,^[18]^ Salmonowicz et al,^[19]^ and Sjögren et al^[27]^ failed to show any relation between duration of diabetes and serum Mg level in type 1 diabetic children. This difference may be due to different study populations and variable degree of diabetic control among these populations.

In our study, there was a significant difference in age between hypomagnesemic and normomagnesemic diabetic children, as mean age of diabetics with low Mg was 4.82 ± 2.35 years versus 1.94 ± 1.45 years in those with normal Mg level (*P* < 0.001). This is agreed with Mishra et al^[22]^ that showed a positive correlation between age of T2DM adults and serum Mg level (*r* = 0.721) (*P* < 0.001) but disagreed with Lin et al^[18]^ and Salmonowicz et al^[19]^ who showed no correlation between age of T1DM children and serum Mg level. Wang et al^[28]^ showed similar results to Lin et al^[18]^ and Salmonowicz et al^[19]^ but in type 2 diabetes patients. Xu et al^[25]^ stated that in adults with T2DM, older age is seen more frequent among normomagnesemic group.

Negative correlation between age of patients and serum Mg level can be attributed to duration of diabetes. This is because most of our patients were diagnosed early in their lives (mean age of diagnosis was 4.21 ± 2.19 with no significant difference between normomagnesemic and hypomagnesemic groups). This was supported by multivariate linear regression which found that age lost its significance while duration of diabetes retained its significance as a predictor of serum Mg.

Our study showed that there was statistically significant difference in serum Mg level before and after Mg supplementation being higher after Mg supplementation with mean ± SD of Mg was 1.45 ± 0.15 mg/dL before Mg supplementation versus 1.94 ± 0.12 mg/dL after Mg supplementation (*P* < 0.001). This is in agreement with Djurhuus et al^[29]^ who revealed increase in serum Mg level with Mg supplementation (500 mg twice daily of MgO) for 24 weeks in adult patients with type 1 diabetes. Also concordant with our results Rodriguez-Moran and Guerrero-Romero^[11]^ in type 2 diabetic adults revealed that there is a significant increase in serum Mg after a period of oral Mg supplementation. However, it is inconsistent with Solati et al^[30]^ who failed to show a significant difference in serum Mg before and after Mg supplementation.

Our study showed that there was a significant difference between HbA1c before and after Mg supplementation as mean value of HbA1c before supplementation was 10.11% ± 0.87% versus 7.88% ± 0.42% after oral supplementation (*P* < 0.001). This indicates that oral Mg level may have a good role on glycemic control of type 1 diabetes. This result is concordant with Rodriguez-Moran and Guerrero-Romero^[11]^ who followed adult type 2 diabetic patients before and after Mg chloride supplementation and showed significant reduction of fasting blood glucose and HbA1c with oral Mg. Moreover, Solati et al^[30]^ showed that oral Mg improves both fasting blood glucose and postprandial blood glucose, but not HbA1c. This double-blind controlled trial was done on adult patients with type 2 diabetes. They used Mg supplement as 300 mg of MgSO_4_ per day. Evidence from epidemiologic studies demonstrated an association between Mg-rich diet and decreased incidence of T1DM and its complications.^[31]^ Dong et al^[32]^ meta-analysis provides further evidence supporting that Mg intake is significantly inversely associated with risk of type 2 diabetes in a dose–response manner. Lopez-Ridaura et al^[33]^ also showed that Mg supplementation decreases the risk of type 2 diabetes in adult population. Indeed, Sales and Pedrosa Lde^[34]^ observed that inadequate metabolic control can affect the corporal concentrations of Mg, developing hypomagnesemia, which may be still directly related with some micro- and macrovascular complications observed in diabetes, as cardiovascular disease, retinopathy, and neuropathy. Based on this, the supplementation with Mg has been suggested in patients with diabetes mellitus who have proven hypomagnesemia and the presence of its complications.

Another supporting meta-analysis was done by Fang et al^[35]^ included 40 prospective cohort studies totaling more than 1 million participants and evaluated the effect of dietary Mg intake on the risk of cardiovascular disease, type 2 diabetes, and all-cause mortality. They concluded that increasing dietary Mg intake is associated with a reduced risk of stroke, heart failure, diabetes, and all-cause mortality, but not coronary heart disease or total cerebrovascular disease.

In disagreement with our results, Djurhuus et al^[29]^ showed reduction in insulin stimulated glucose uptake after Mg supplementation in adult type 1 diabetic patients but did not observe any changes in glycemic control or dose of insulin with oral Mg. Also Lal et al^[36]^ studied patients of T2DM and found no significant difference in glycemic control before and after supplementation with 600 mg of Mg oxide daily for 12 weeks, this difference in results may be explained by different study populations and type of diabetes between them and ours. Meta-analysis was done by Simental-Mendía et al^[37]^ to observe the effect of oral Mg supplementation on insulin sensitivity and glucose control in both diabetic and nondiabetic individuals. It said that a significant effect of Mg supplementation was observed on Homeostatic Model Assessment and Insulin Resistance index, but not on plasma glucose, HbA1c, and insulin.

In our study, we detected a significant difference in lipid parameters before and after oral Mg supplementation in hypomagnesemic diabetic children with lower value of TC, TG, and LDL after supplementation and higher HDL after supplementation (*P* < 0.001). This is in agreement with Djurhuus et al^[29]^ that observed decreased atherogenic lipid fractions (total cholesterol, LDL, and Apo lipoprotein B), with Mg supplementation in type 1 diabetes and reduced the risk of cardiovascular complications. Also Lal et al^[36]^ observed a significant fall in serum total cholesterol, LDL, and triglycerides and a rise in HDL 4 to 8 weeks after Mg supplementation. Consistent with our study also Solati et al^[30]^ showed a significant reduction in LDL in Mg-treated diabetic patients.

## 5 Conclusion

Our study demonstrates that serum total Mg is frequently low in Egyptian children with type 1 diabetes, and it is correlated with glycemic control and lipid profile. Also we concluded that correction of hypomagnesemia in type 1 diabetic children with oral Mg supplements is associated with optimization of glycemic control and reduction of atherogenic lipid fraction as well as increase in protective lipid fraction.

## 6 Limitations

There are some limitations in our study like limited number of patients, limited resources that enabled us only to focus on serum Mg level, glycemic control, and lipid profile but did not allow us to measure other metabolic parameters that would help us like adiponectin, leptin, and insulin like growth factor 1. We recommend performing further multicenter studies on different populations with different levels of glycemic control to support our findings.

## Acknowledgments

The authors thank all the participants of this study for their unstinted cooperation. Authors also thank Dr Mohamed Fathy, lecturer of oncology medicine and statistics for his great help in performing the statistics of this work.
